# Advances in research on potential therapeutic approaches for Niemann-Pick C1 disease

**DOI:** 10.3389/fphar.2024.1465872

**Published:** 2024-08-28

**Authors:** Caifeng Zhang, Keke Su, Xu Jiang, Yuping Tian, Ke Li

**Affiliations:** ^1^ Department of Gastroenterology, The First Affiliated Hospital of Xinxiang Medical University, Xinxiang, China; ^2^ First College for Clinical Medicine, Xinxiang Medical University, Xinxiang, Henan, China

**Keywords:** cell-based therapy, combination therapy, small molecule therapy, disease models, gene therapy, Niemann-Pick disease type C1

## Abstract

Niemann-Pick disease type C1 (NP-C1) is a rare and devastating recessive inherited lysosomal lipid and cholesterol storage disorder caused by mutations in the NPC1 or NPC2 gene. These two proteins bind to cholesterol and cooperate in endosomal cholesterol transport. Characteristic clinical manifestations of NP-C1 include hepatosplenomegaly, progressive neurodegeneration, and ataxia. While the rarity of NP-C1 presents a significant obstacle to progress, researchers have developed numerous potential therapeutic approaches over the past two decades to address this condition. Various methods have been proposed and continuously improved to slow the progression of NP-C1, although they are currently at an animal or clinical experimental stage. This overview of NP-C1 therapy will delve into different theoretical treatment strategies, such as small molecule therapies, cell-based approaches, and gene therapy, highlighting the complex therapeutic challenges associated with this disorder.

## Introduction

Niemann-Pick type C1 disease (NP-C1) was first described in the early twentieth century and further elucidated by Alan Crocker in the 1960s (Crocker, 1961). Peter Pentchev, two decades later, conducted a series of investigations on a spontaneous mouse model, shedding light on the molecular processes underlying this enigmatic disorder (Pentchev et al., 1986). NP-C1 is now recognized as a prototypical lysosomal storage disorder, characterized by the abnormal accumulation of cholesterol and other lipids in late endosomes and lysosomes (LE/LY) (Brauer et al., 2019). While lipid buildup is observed in various tissues such as the liver, spleen, lungs, and bone marrow, the most critical disease manifestations stem from progressive neurodegeneration (Vanier, 2010; Patterson and Walkley, 2017).

The NP-C1 is a rare condition with a worldwide estimated incidence ranging from 1/1,00,000 to 1/1,20,000 live births (Mengel et al., 2013). However, the actual incidence is likely higher due to frequent misdiagnoses or cases that go undetected (Stampfer et al., 2013). NP-C1 presents with varying ages of onset, categorized into infancy, childhood, juvenile, adolescence, and adulthood. Most patients experience symptoms in early childhood and typically pass away within 5–20 years after the onset of the disease (Hung et al., 2014). The clinical diagnosis of NP-C1 currently relies on laboratory analyses, including filipin staining of skin fibroblasts and biomarker assessments. However, a definitive diagnosis is confirmed through next-generation sequencing (NGS) analysis (Encarnacao et al., 2023). Patients with NP-C1 typically present with liver and spleen enlargement in the early stages, followed by progressive neurodegeneration and other symptoms as the disease advances (Nadjar et al., 2018). Unfortunately, there are limited well-established pharmacological treatments available for NP-C1. Over the past two decades, researchers have explored diverse approaches for managing NP-C1 through animal or clinical experiments (Patterson and Platt, 2004). This review aims to provide a comprehensive overview of NP-C disease, delving into various treatment strategies from multiple angles, including traditional small molecules, cell-based therapies, and gene therapy. The discussion highlights the intricate therapeutic challenges associated with combating this debilitating condition.

## NPC protein function

Cholesterol serves multiple crucial functions in the body, acting as a key component of cell membranes and plasma lipoproteins while also serving as a precursor for bile acids, hormones, and vitamin D3. As such, the synthesis and transport of cholesterol are essential for maintaining cellular integrity (Lamri et al., 2018). In NP-C1, there is a disruption in the intracellular transport and balance of free cholesterol. The accumulation of excess free cholesterol in late endosomes and lysosomes can lead to significant cellular and tissue damage in the nervous system and other organs (Lopez et al., 2014). NP-C1 in humans is primarily caused by mutations in two genes, NPC1 and NPC2, with approximately 95% of cases linked to NPC1 mutations and 5% to NPC2 mutations (Vanier, 2010).

NPC1 is a large transmembrane protein located on the boundary membrane of late endosomes and lysosomes, consisting of 13 transmembrane domains encoding 1,278 amino acids crucial for intracellular cholesterol transport. On the other hand, NPC2 is a soluble lumen protein responsible for transporting cholesterol from lysosomal vesicles to the N-terminal domain of NPC1. Both proteins are essential for cholesterol export from lysosomes (Naureckiene et al., 2000; Infante et al., 2008a; Scott and Ioannou, 2004). While the precise roles of NPC1 and NPC2 in lysosomal cholesterol transport are not fully understood, it is believed that they are involved in pathways regulating cholesterol transport within lysosomes or promoting movement of lysosomal lipid substrates (Lloyd-Evans and Platt, 2010; Infante et al., 2008b).

**FIGURE 1:**
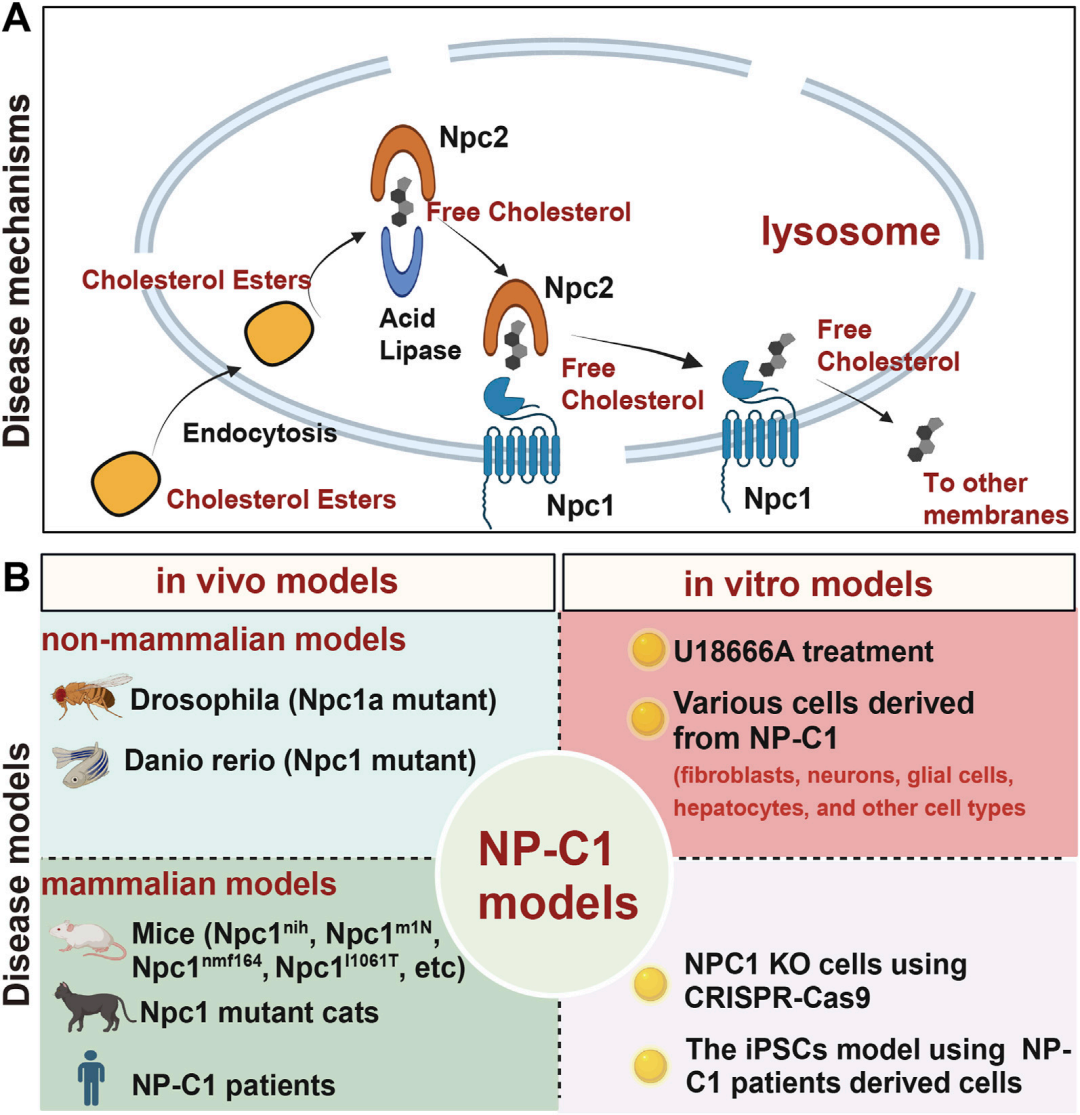
A brief overview of the mechanisms and models of NP-C1. **(A)** The process by which NPC1 and NPC2 proteins transport cholesterol from lysosomes to other organelle membranes. **(B)** Current *in vivo* and *in vitro* models used for researching NP-C1.

A prevailing hypothesis suggests that when a lipid cargo reaches late endosomes/lysosomes, it is broken down into its constituent molecules. NPC2 facilitates the transfer of cholesterol or other lipids to organelle boundary membranes, while NPC1 detects an increase in cholesterol at the cell membrane and initiates transport of the cargo to its designated destinations (Wang et al., 2010; Kwon et al., 2009). NPC1, working in conjunction with NPC2, facilitates the removal of low-density lipoprotein (LDL)-derived cholesterol from the endosomal compartment through a yet-to-be-defined mechanism, although significant progress has been achieved (Somers et al., 2003). The generally accepted model posits that NPC2 binds free cholesterol post-hydrolysis of LDL cholesterol esterase and transfers it to NPC1, which then mediates the extraction of cholesterol from the lysosome (Zampieri et al., 2014) (Figure 1A). Consequently, the absence of either NPC1 or NPC2 protein could lead to the manifestation of this genetic disease, as there are no substitutes for these two proteins in NP-C1.

## Currently used NP-C1 model systems

The clinical spectrum of NP-C1 varies from a severe neonatal disorder to a chronic neurodegenerative disease that can onset in adulthood. The most severe cases present in early childhood and result in death during childhood or adolescence. The onset of NP-C1 symptoms can vary among individuals, with some experiencing disease manifestation in adolescence or adulthood due to different genetic information influencing disease progression. Given ethical considerations, the timing of symptom onset, and the complexities involved, the development of animal and cell models is essential for studying NP-C1 and advancing our understanding of this disorder (Figure 1B). The evolving NP-C1 models should offer several advantages, including ease of gene manipulation, straightforward modification and screening processes, and are particularly well-suited for establishing platforms for the discovery and development of high-throughput drugs.

### *In vivo* models

Numerous animal models have been employed in the study of NP-C1, owing to the relatively conserved nature of the genes and functions of Npc1 across non-mammalian and mammalian populations. A disease model has been developed by mutating *Drosophila Npc1a*. Null mutants exhibit early lethality, movement impairments, neuronal cholesterol deposits, accumulation of multilamellar bodies, and age-dependent neurodegeneration that mimics the human neurodegenerative condition (Phillips et al., 2008). In zebrafish (*Danio rerio*), *Npc1* morphants and mutants show high lethality, reduced steroidogenesis, abnormal cell movements, and a severe neurometabolic phenotype (Quelle-Regaldie et al., 2023; Schwend et al., 2011). In addition, the nematode *Caenorhabditis elegans* and the yeast *Saccharomyces cerevisiae* have been developed and utilized to gain insights into NPC cellular pathways (Boland et al., 2017; Frain et al., 2024). Currently, investigations into non-mammalian NP-C1 predominantly concentrate on *Drosophila* and *D. rerio*. Nevertheless, research in this area has slowed in recent years, largely due to the rapid advancement of mammalian and *in vitro* models.

Studies on NP-C1 in mammals predominantly focus on mice and cats, which are frequently used in laboratory research and clinical drug development studies. Different mutations in the Npc1 gene in mice can lead to varying phenotypes. These mice are currently the most common NPC1 rodent model for assessing potential small molecules for this debilitating disease. The initial mouse strains used for studying NP-C1, namely *C57BLKS/J* ^*spm*^ and *BALB/cNpc1* ^*nih*^, originated as spontaneous mutations. Through crossbreeding, researchers confirmed them as allelic and independently positioned them on mouse chromosome 18, in a region syntenic to the human NPC1 locus (Loftus et al., 1997; Pentchev et al., 1984). These two commonly used mice models display typical NP-C1 neurological symptoms such as hepatomegaly, splenomegaly, decreased weight gain, increased lung mass, disturbed motor coordination, tremor, ataxia and loss of Purkinje cells (Loftus et al., 1997). The pathology of NP-C1 in these mouse models initiates at 4–6 weeks of age, closely resembling the onset of NP-C1 in infants and young children. They serve as valuable research tools for studying the severe infantile onset forms of NP-C1.

Maue et al. established a new mouse model (*Npc1* ^*nmf164*^) with a point mutation in the Npc1 gene, specifically an A to G change at cDNA base pair 3,163, resulting in an aspartate to glycine substitution at position 1005 (D1005G). The lifespan analyses show that these mice begin developing the disease at 4 weeks and typically survive until around 16 weeks, indicating a lifespan extension of approximately 5 weeks compared to *Npc1* ^*nih*^ mice. The histological analyses reveal abnormal cholesterol accumulation, glial activation and Purkinje cell loss at a slower rate than in the *Npc1* ^*nih*^ mouse model (Maue et al., 2012). *Praggastis* and colleagues developed an *Npc1* ^*I1061T*^ knock-in mouse model that shows a less severe and delayed form of NP-C1. This model is characterized by reduced weight loss, improved motor coordination, decreased Purkinje cell death, reduced lipid storage, and delayed premature death compared to the *Npc1* ^*nih*^ mice (Praggastis et al., 2015). Meanwhile, the lifespan analyses show that these mice begin developing the disease at 8 weeks and pass away within 17–18 weeks. Taken together, these two mice models offer many advantages as a model for the late-onset, more slowly progressing forms of NP-C1 that comprise the large majority of human cases. Furthermore, current research is exploring the effects of cholesterol transport abnormalities on organ development and homeostasis through using the *Cre-loxP* system to selectively target *Npc1* gene in specific tissues (Elrick et al., 2010). NP-C1 in humans is a genetic disorder commonly associated with widespread organ dysfunction. Therefore, mice with systemic *Npc1* mutations are considered more appropriate candidates for NP-C1 drug development. However, different mutations in Npc1 mutant mice can result in varying phenotypes, reflecting the complexity observed in clinical cases. These diverse mutations in the NPC1 gene result in the production of unique proteins, triggering a cascade of biochemical responses in the body and giving rise to a spectrum of disease presentations.

Additionally, a feline model of NP-C1 has been meticulously characterized, exhibiting phenotypic, morphological, and biochemical similarities to human NPC1. The disease manifestation in NP-C1 felines mirrors the juvenile form of human NP-C1, replicating numerous clinical features such as hepatomegaly, pulmonary complications, and central nervous system (CNS) involvement, notably ataxia, among other symptoms (Rakib et al., 2023). Research on *Npc2* gene mutations causing NP-C1 is relatively limited compared to studies on the *Npc1* gene. However, in mouse and cat models, mutations in the *Npc2* gene have been shown to result in symptoms similar to those observed in clinical NP-C1, including weight loss, decreased motor coordination, cerebellar Purkinje neuron death, lipid storage, and premature death (Rakib et al., 2023; Lee and Hong, 2023; Pallottini and Pfrieger, 2020).

### *In vitro* models

In addition to the NP-C1 animal models mentioned above, researchers have developed cell models for NP-C1, emphasizing the direct role of lysosomal lipid accumulation in cellular signal transduction and phenotype analysis. Researchers isolated fibroblasts, neurons, glial cells, hepatocytes, and other cell types from *Npc1* mutant mice with diverse genotypes to investigate cellular states throughout the progression of NP-C1 (Peake and Vance, 2012; Malara et al., 2024; Kulinski and Vance, 2007). Findings from these studies using cells from mutant mice suggest that the absence of the *Npc1* disrupts cholesterol metabolism, impacting intracellular processes such as autophagy, endoplasmic reticulum (ER), vesicle sorting, and multiple signaling pathways (Hoglinger et al., 2019; Schwerd et al., 2017; Sarkar et al., 2013).

In humans, obtaining a comprehensive understanding of the majority of NP-C1 variants using patient-derived fibroblasts remains challenging due to their infrequent occurrence in isolation. Recent advances in induced pluripotent stem cells (iPSCs) technology have made it possible to create cell-based disease models using human cells derived from patient iPSCs. Utilizing a fibroblast-induced iPSCs model from NP-C1 patients can offer valuable insights into the pathological mechanisms of NP-C1 (Trilck et al., 2013). The iPSCs derived from NP-C1 fibroblasts express various stem cell markers, differentiate into cells of all three germ layers, and induce teratoma formation in immunodeficient mice. Additionally, the iPSCs from NP-C1 patients exhibit cholesterol accumulation in the cytoplasm, a characteristic not seen in cells from healthy individuals. These observations demonstrate that iPSCs derived from patient cells retain pluripotency while displaying disease-specific features, highlighting their potential as a valuable tool for studying and modeling diseases (Trilck et al., 2013; Volkner et al., 2022). While the iPSCs model established using fibroblasts provides valuable insights into NP-C1, it may be limited in studying organ-specific pathologies such as neuronal loss and hepatocyte damage. Therefore, it is crucial to differentiate the iPSCs into specific cell types like neurons, glial cells and hepatocyte-like cells using specialized methods during *in vitro* modeling. These approaches are essential for gaining a deeper understanding of the mechanisms underlying brain and liver dysfunction in NP-C1 (Prabhu et al., 2021; Volkner et al., 2021; Peter et al., 2017).

The use of CRISPR/Cas9 technology to knockout specific genes within the genome of mammals has indeed become routine in recent years (Du et al., 2017). Research has indeed shown that knocking out the *Npc1* gene using technology can lead to cholesterol accumulation characteristics in various cells. Deletion of the *Npc1* has been associated with enhanced cell connectivity in 293T cells and may promote inflammation response and apoptosis in N2a cells (Du et al., 2017; Jia et al., 2023; Yang et al., 2023). Furthermore, researchers employed the saturation prime editing (SPE) platform to edit the *Npc1* gene, revealing that 410 out of the 706 assayed missense mutations present significant risks for the disease (Erwood et al., 2022). Additionally, a haploid cell model established using CRISPR/Cas9 technology serves as a valuable platform for studying the pathogenesis of NP-C1 (Erwood et al., 2019). These findings highlight the importance of *Npc1* in cellular function and the significant potential of gene editing technology in developing NPC1 models. With the advancement of CRISPR/Cas9 technology, achieving single-base mutations has become feasible (Chen and Liu, 2023). By integrating CRISPR/Cas9 with iPSCs technology (Xu et al., 2019), a wide range of NP-C1 cell models will be generated. This potent combination enables accurate genetic alterations, rendering it an invaluable asset for exploring gene functionality and disease mechanisms across diverse organisms.

## Potential therapeutic approaches for NP-C1

Currently, there are limited effective therapies for NP-C1. Treatment mainly focuses on symptom management and slowing down the progression of the disease. Some treatment methods include drug therapy, nutritional support and physical therapy, aiming to improve the quality of life and delay the progression of the disease. In recent years, advancements in NP-C1 animal and cell models have provided a more profound insight into the pathogenesis of NP-C1, facilitating high-throughput drug screening. The introduction of innovative technologies like gene editing and stem cell therapy has expanded the scope of potential treatment options for the disease. Nevertheless, additional research and clinical trials are imperative to pinpoint more effective treatment strategies.

## Small molecule therapeutics

Indeed, as our understanding of the molecular mechanisms of NP-C1 has improved, researchers have been able to explore a variety of small molecules for treating the condition. By conducting studies and clinical trials, researchers have made significant progress in developing treatments for NP-C1. These efforts are directed towards offering improved management and potential therapeutic options for individuals affected by NP-C1 (Table 1).

**TABLE 1:** Small molecule therapeutics in NP-C1 treatment.

Small molecules	Models	Effects	Reference
Statins	*Npc1-KO* and U18666A SH-SY5Y cells	Reverse intracellular cholesterol accumulation, decreased α-synuclein aggregation and secretion	Min et al. (2023)
Simvastatin	*Npc1*-deficient macrophagy	Promote Npc1-mediated free cholesterol efflux from lysosomes through CYP7A1/LXRα pathway	Xu et al. (2017)
Lovastatin	*Npc1* ^*−/−*^ mouse and oligodendrocytes	Enhances the number of mature myelin-forming oligodendrocytes by increasing Olig1 and Olig2 expressions	Yang et al. (2018)
Statins	*Npc1* ^*−/−*^ neurons	Statin treatment may endanger survival of cells by interfering in the autophagy	Meske et al. (2019)
Miglustat	*Npc1* ^*nih*^ mice and *Npc1* ^*−/−*^ patients	Modestly decreased gangliosides in liver and brain in Npc1nih mice; alterations in plasma gangliosides and CSF sphingolipids in patients	Fan et al. (2013)
Miglustat	*Npc1* ^*m1N*^ mice and neurons	Increase the expression of Flot2 in *Npc1* ^*m1N*^ mice and neurons	Chen et al. (2023)
Miglustat	*Npc1* ^*−/−*^ cats	Delayed the onset of neurological signs; increased the lifespan; decreased ganglioside accumulation; improved purkinje cell survival	Stein et al. (2012)
Miglustat	*Npc1* ^*nih*^ mice	Rescue synaptic plasticity deficits, restore ERKs activation; counteract hyperexcitability	D’Arcangelo et al. (2016)
Miglustat	*Npc1* ^*−/−*^ patients and *Npc1* ^*I1061T*^ mice	Decreased the NPY levels	Li et al. (2023)
Miglustat	*Npc1* ^*−/−*^ patients	Sabilize neurological manifestations in late-infantile and juvenile-onset forms of NP-C1 rather than infantile-onset NP-C1	Nadjar et al. (2018); Di Rocco et al. (2012); Heron et al. (2012)
Miglustat	*Npc1* ^*−/−*^ patients	Stabilized swallowing function and reduced aspiration risk in NP-C1	Solomon et al. (2020)
HPβCD	*Npc1* ^*−/−*^ mice *Npc2* ^*−/−*^ mice	Decreased expression of proinflammatory proteins; improved in liver function; less neurodegeneration; prolongation of life span	Lopez et al. (2014); Liu et al. (2009); Davidson et al. (2009)
HPβCD	*Npc1* ^*−/−*^ mice and oligodendrocytes	rescue myelination, epigenetic marks, and oligodendrocyte gene expression	Kunkel et al. (2023)
HPβCD	*Npc1* ^*−/−*^ mice *Npc1* ^*−/−*^ cats	Enhanced purkinje cell survival and reversed all microglia-associated defects	Marschalek et al. (2014); Vite et al. (2015); Cougnoux et al. (2018a); Barthelemy et al. (2021)
HPβCD	*Npc1* ^*−/−*^ mice	Biomarkers for therapeutics: cathepsin S in the liver, 24(S)-hydroxycholesterol in serum, and calbindin D in CSF	Alam et al. (2014); Jiang et al. (2014); Tortelli et al. (2014); Bradbury et al. (2016)
HPβCD	*Npc1* ^*−/−*^ patients *Npc1* ^*−/−*^ mice	GPNMB as a biomarker for therapeutics	Rodriguez-Gil et al. (2021); Fukaura et al. (2021)
HPβCD	*Npc1* ^*−/−*^ mice	No effect on the progressive pulmonary disease	Ramirez et al. (2010); Muralidhar et al. (2011)
HPβCD	*Npc1* ^*nmf164*^ mice	Increased inflammatory response in lung	Erickson et al. (2018)
HPβCD	*Npc1* ^*−/−*^ neurons and glial cells	Extracts cholesterol from the plasma membrane and reduces ER cholesterol	Peake and Vance (2012)
HPβCD HPγCD	*Npc1* ^*−/−*^ fibroblasts	LAMP-1 rescue the cholesterol accumulation in NP-C1	Singhal et al. (2018)
HPβCD	*Npc1* ^*−/−*^ mice, neurons and axons	Rescue lysosome transport, reduce axonal autophagic stress and neuron death	Roney et al. (2021)
HPβCD	*Npc1* ^*−/−*^ fibroblasts	Increased the number of LC3-positive puncta and the levels of p62	Tamura and Yui (2015)
MβCD	*Npc1* ^*−/−*^ fibroblasts	Restored impaired autophagy flux, activated AMPK pathway	Dai et al. (2017)
HPβCD	*Npc1* ^*−/−*^ mice *Npc1* ^*−/−*^ cats	Caused a significant increase in hearing threshold	Ward et al. (2010)
HPβCD	*Npc1* ^*−/−*^ mice Adult rats	Caused irreversible hearing loss; both inner and outer hair cell death	Takahashi et al. (2016); Zhou et al. (2018); Liu et al. (2020)
HPβCD	Adult rats	Destroyed both outer and inner hair cells, auditory nerve fibers, spiral ganglion neurons and vestibular ganglion neurons	Ding et al. (2020)
Vorinostat (HDAC-1,2,3,6)	*Npc1* ^*−/−*^ fibroblasts	Significantly lowered the relative amount of unesterified cellular cholesterol	Wehrmann et al. (2012); Helquist et al. (2013); Pipalia et al. (2017)
Vorinostat (HDAC-1,2,3,6)	*Npc1* ^*nmf164*^ mice	Reduce the cellular cholesterol levels; did not improve animal survival	Alam et al. (2016)
Vorinostat (HDAC-1,2,3,6)	*Npc1* ^*nmf164*^ mice and *Npc1* ^*−/−*^ hepatocytes	Refolding of Npc1 mutant protein; modulates apoB metabolism; improves liver function; does not delay weight loss	Munkacsi et al. (2017)
Panobinostat (HDAC-1,2,3,6)	*Npc1* ^*−/−*^ fibroblasts	Reduce the cellular cholesterol levels and restore cholesterol homeostasis	Wehrmann et al. (2012); Pipalia et al. (2011); Pipalia et al. (2017)
Valproic acid (HDAC-1,2,6)	*Npc1* ^*−/−*^ NSCs	Enhance neuronal differentiation and recover defective cholesterol metabolism	Kim et al. (2007)
Valproic acid (HDAC-1,2,6)	*Npc1* ^*I1061T*^ patient fibroblasts	Reduce cholesterol accumulation; enhance NPC1-I1061T expression and trafficking; reduce HDAC7 expression	Subramanian et al. (2020)
Curcumin	*Npc1* ^*−/−*^ mice	Improve NP-C1 cellular phenotypes; prolong survival of the NPC1 mouse; regulate cytosolic calcium levels	Lloyd-Evans et al. (2008)
Nicotinamide	*Npc1* ^*−/−*^ mice	Increased survival; reversing oxidative stress	Marshall et al. (2017)
FTY720	*Npc1* ^*−/−*^ fibroblasts	Increase the expression of NPC1; reduce the accumulation of cholesterol and GSLs	Newton et al. (2017)
HNHA	*NPC1-iNSCs*	Improve body weight and motor function; Reduce purkinje cell death; Increase the protein levels of SNAP25; Induce autophagy	Jung et al. (2022)
Arimoclomol/rhHSP70	*Npc1* ^*−/−*^ mice	Reduce GSLs levels; improve cerebellar myelination and behavioural phenotypes	Gray et al. (2022)
HSP90 inhibitors	*Npc1* ^*I1061T*^ fibroblasts	Increase HSP70 levels; promote the cholesterol trafficking; reduce cholesterol storage	Pipalia et al. (2021)
Imatinib (c-Abl inhibitor)	*Npc1* ^*−/−*^ mice	Reduce purkinje neurons; improve neurological symptoms; increase survival of *Npc1* ^*−/−*^ mice	Alvarez et al. (2008)
Gadolinium chloride	*Npc1* ^*−/−*^ mice	Decrease the CD68 positive cells; normalize the transaminase levels; rescue the liver dysfunction	Klein et al. (2018)
Pneumococci	*Npc1* ^*−/−*^ mice	Improve liver lipid accumulation and inflammation; improve cerebellar phenotype and neuroinflammation; delay the regression of motor skills	Houben et al. (2018)
Necrostatin-1	*Npc1* ^*−/−*^ mice *Npc1* ^*−/−*^ fibroblasts	Delay cerebellar purkinje cell loss; improve neurological symptoms	Cougnoux et al. (2016)
LXR agonist	*Npc1* ^*−/−*^ mice	Increase cholesterol excretion; decrease neuroinflammation; deactivation of microglia; extend the lifespan	Repa et al. (2007)
BK channel agonist	*Npc1* ^*−/−*^ fibroblasts	Reduce lipofuscin and cholesterol accumulation	Cao et al. (2015); Zhong et al. (2016)
Lithium carbonate	*Npc1* ^*−/−*^ patients	Improve swallowing capacity	Han et al. (2021)
Lithium	*Npc1* ^*−/−*^ mice *Npc1* ^*I1061T*^ mice	Improve ataxia and feeding phenotypes; attenuate cerebellar inflammation and degeneration; extends survival	Han et al. (2023)
GSH ethyl ester	*Npc1* ^*−/−*^ mice	Improve oxidative phosphorylation; protect against oxidative stress; restore purkinje cells; reverse locomotor impairment; increased the lifespan	Torres et al. (2017)
S-adenosyl-L-methionine	*Npc1* ^*−/−*^ mice	Improve the decline of locomotor activity; increase purkinje cell survival; extend the average and maximal lifespan	Goicoechea et al. (2024)
Genistein	*NP-C1* patient fibroblasts	Reduce p62 levels and increase levels of LC3-II	Arguello et al. (2021)

### Statins

Statins, 3-hydroxy-3-methylglutaryl-coenzyme A reductase inhibitors, are a vital class of drugs used to lower cholesterol levels. Researchers are investigating the potential therapeutic benefits of statins for NP-C1, as they believe that these drugs may have a positive impact on the accumulation and impaired trafficking of cholesterol in *Npc1*-deficient cells. Studies have demonstrated that statins can reverse intracellular cholesterol accumulation and α-synuclein aggregation induced by *Npc1*-KO or U18666A treatment (Min et al., 2023). In macrophages, simvastatin promotes Npc1-mediated free cholesterol efflux from lysosomes through CYP7A1/LXRα pathway (Xu et al., 2017). While lovastatin has been shown to restore the number of mature myelin-forming oligodendrocytes (Yang et al., 2018), Meske et al. reported that statin treatment may pose a risk to the survival of Npc1 mutant neurons (Meske et al., 2019). These findings suggest that statin drugs may have the potential to improve symptoms in NP-C1 due to their cholesterol-lowering properties, but they do not seem to effectively alter the neurological progression.

### Miglustat

In addition to the cholesterol accumulation, *Npc1*-deficient cells also accumulate gangliosides and other glycosphingolipids (GSLs), which are key players in the pathogenesis of NPC disease (Zervas et al., 2001). Miglustat, a reversible inhibitor of GSLs synthesis recognized for its ability to reduce GSLs buildup in type 1 Gaucher’s disease, has demonstrated positive effects through reducing GSLs levels on the progression of NP-C1 (Pineda et al., 2018). In addition to GSLs, there was a notable accumulation of gangliosides in the liver and brain. Following miglustat treatment in NP-C1 animal models and NP-C1 patients, the levels of gangliosides in plasma and cerebrospinal fluid (CSF) decreased significantly (Fan et al., 2013). In *Npc1* ^−/−^ mice, gangliosides sequestration and the loss of lipid rafts lead to cell dysfunction and symptoms of NP-C1. Miglustat increased the expression of Flot2, a marker for lipid rafts, which was found to be diminished in neurons of *Npc1* ^−/−^ mice (Chen et al., 2023).

Accumulation of gangliosides and GSLs in neurons is closely linked to cell metabolism, maintaining homeostasis, and cell death. Research in the NPC1 feline disease model indicates that miglustat delayed the onset of neurological symptoms, extended the lifespan of treated cats, and correlated with reduced ganglioside buildup in the cerebellum and enhanced Purkinje cell survival. Analyzing microglia from treated cats decreased production of reactive oxygen species (ROS) (Stein et al., 2012). Furthermore, the administration of miglustat in *Npc1* ^*−/−*^ mice demonstrated the ability to rescue deficits in synaptic plasticity, restore ERK activation, and alleviate hyperexcitability (D’Arcangelo et al., 2016). Collectively, these results indicate that the prolonged survival of Purkinje cells, reduction in ganglioside accumulation, and restored synaptic plasticity are key factors contributing to the neurological enhancements observed in NPC-1 individuals treated with miglustat.

Intense inflammation is a significant factor that triggers multi-organ damage in NP-C1. Miglustat has been shown to suppress astrocyte pathogenic activities in CNS inflammation by inhibiting the production of pro-inflammatory cytokines and restoring lactate generation in astrocytes (Chao et al., 2019). While there are currently few specific data confirming the anti-inflammatory role of miglustat in NPC1, several studies have confirmed its combined use with anti-inflammatory drugs in this condition. Pro-neuropeptide Y (NPY) levels were significantly elevated in individuals with NPC1 compared to healthy controls. Miglustat demonstrated efficacy in mitigating neuroinflammation and decreasing excitotoxicity by modulating NPY levels (Li et al., 2023).

For NP-C1 with varying onset periods, research indicates that miglustat can stabilize neurological manifestations in pediatric patients with late-infantile and juvenile-onset forms of NP-C1, rather than specifically targeting neurologic manifestations in infantile-onset NP-C1 (Di Rocco et al., 2012; Heron et al., 2012). Adolescent/adult-onset NP-C1 often initially presents with non-specific isolated neuro-psychiatric manifestations (motor, cognitive, or psychotic). Patients with milder neurological disabilities tend to respond more positively to miglustat therapy (Nadjar et al., 2018). Furthermore, the use of miglustat is linked to stabilized swallowing function and reduced aspiration risk in NP-C1, underscoring the potential for quantifying swallowing dysfunction as a clinically relevant functional outcome measure in future therapeutic trials for NP-C1 (Solomon et al., 2020).

### 2-hydroxypropyl--cyclodextrin (HPβCD)

Miglustat has shown limited effectiveness in reducing substrates and does not affect the accumulation of cholesterol in the body. Furthermore, its ability to slow the progression of neurological symptoms is also constrained (Pineda et al., 2018). Cyclodextrins (CDs), a family of cyclic oligosaccharides, have the ability to form complexes with cholesterol, effectively replacing dysfunctional cholesterol transport proteins. In NP-C1, 2-hydroxypropyl-β-cyclodextrin (HPβCD) is being considered as an alternative treatment option. A range of studies have demonstrated the effectiveness of HPβCD in treating NP-C1. A single dose of HPβCD has been shown to suppress sterol synthesis, down-regulate SREBP2 and its target genes, and reduce the expression of macrophage-associated inflammatory genes in the liver and brain (Liu et al., 2009; Liu et al., 2010). Additionally, HPβCD was able to rescue myelination, epigenetic marks, and oligodendrocyte gene expression, emphasizing the critical role of HPβCD in oligodendrocyte lineage maturation and epigenetic regulation in NP-C1 (Kunkel et al., 2023). Prolonged administration of HPβCD has shown improvements in liver function tests, reduced neurodegeneration, amelioration of cholesterol or GSLs storage, and a significant extension of lifespan (Lopez et al., 2014; Liu et al., 2009; Liu et al., 2010; Davidson et al., 2009). Importantly, treatment with HPβCD resulted in a reduction in Purkinje cell loss, reversed microglia-mediated neuroinflammation, and induced a concerted action of neurons and glial cells to restore lipid homeostasis in the CNS (Marschalek et al., 2014; Vite et al., 2015; Cougnoux et al., 2018a; Barthelemy et al., 2021). Several biomarkers in serum and body fluids have been developed for predicting the prognosis of NP-C1 with HPβCD treatment. These include cathepsin S in the liver, 24(S)-hydroxycholesterol in serum, and Calbindin D in CSF (Alam et al., 2014; Jiang et al., 2014; Tortelli et al., 2014; Bradbury et al., 2016). Transcriptome sequencing analysis of NPC1 patients receiving cyclodextrin therapy identified GPNMB as a key factor in evaluating treatment efficacy (Rodriguez-Gil et al., 2021; Fukaura et al., 2021). While HPβCD treatment shows significant protective effects in the brain and liver in NP-C1, its influence on lung dysfunction is minimal (Ramirez et al., 2010; Muralidhar et al., 2011; Erickson et al., 2018). This implies that lung cells may have the capability to resist the effects of CDs on cholesterol trafficking.

A mutation in NPC1 leads to the sequestration of unesterified cholesterol in the late endosomal/lysosomal compartment. HPβCD binds to unesterified cholesterol and facilitates its delivery from the lysosome to the ER, resulting in a significant increase in ACAT-mediated cholesterol esterification and a decrease in unesterified cholesterol levels (Liu et al., 2009; Aqul et al., 2011). The accumulation of cholesteryl esters in the cytosol is anticipated to be considerably less toxic than the accumulation of free cholesterol in NP-C1 patients (Abi-Mosleh et al., 2009). LAMP1, situated in the membranes of lysosomes, was upregulated in response to HPβCD treatment. This upregulation facilitated cholesterol trafficking at the late endosome/lysosome compartments, effectively rescuing the cholesterol accumulation defect observed in fibroblast cells derived from NPC1 patients (Singhal et al., 2018). On the other hand, HPβCD treatment significantly increased autophagic flux and restored lysosome transport, leading to a reduction in axonal autophagic stress and neuronal death in NP-C1 (Roney et al., 2021; Tamura and Yui, 2015) Methyl-β-cyclodextrin (MβCD), a potent analog of HPβCD, was also found to restore impaired macroautophagy/autophagy flux in NP-C1 by activating the AMPK pathway (Dai et al., 2017). These findings demonstrate the translational promise of HPβCD in enhancing impaired autophagic flux and reinstating axonal homeostasis in the initial phases of NP-C1.

### HDAC inhibitors (HDACi)

Histone deacetylase inhibitors (HDACi) are emerging as promising therapeutics for a diverse array of diseases, encompassing cancer and neurodegenerative conditions. Recently, a genome-wide, conditional synthetic lethality screen was conducted using the yeast model of NP-C1 (Munkacsi et al., 2011). Additionally, a high-content screen targeting the reduction of lysosomal cholesterol storage was performed with *Npc1* ^*1I1061T*^ patient fibroblasts (Pugach et al., 2018). Both studies suggest that HDACi emerges as a promising candidate therapy for NP-C1.

Two HDAC inhibitors, vorinostat and panobinostat, have been reported to be effective in treating various cancers and are showing promise in the management of cholesterol metabolism disorders. Both vorinostat and panobinostat have demonstrated the ability to reduce levels of unesterified cellular cholesterol and restore cholesterol homeostasis in *Npc1* ^*−/−*^ fibroblasts (Wehrmann et al., 2012; Helquist et al., 2013; Pipalia et al., 2011). Vorinostat additionally improves liver function and modulates apoB metabolism, although it does not delay weight loss or enhance animal survival in *Npc1* ^*nmf164*^ mice (Alam et al., 2016; Munkacsi et al., 2017). Mechanistically, vorinostat and panobinostat enhance the expression and trafficking of the NPC1 mutant protein, revealing unanticipated epigenomic plasticity in spatial covariance relationships that restore NPC1 functionality (Pipalia et al., 2017; Wang et al., 2019). Valproic acid (VPA), a histone deacetylase inhibitor, has the potential to promote neuronal differentiation and restore impaired cholesterol metabolism in neural stem cells (NSCs) derived from *Npc1*-deficient mice (Kim et al., 2007). In *Npc1* ^*1I1061T*^ models, VPA enhances NPC1-I1061T protein expression and trafficking, resulting in the restoration of cholesterol homeostasis by reducing HDAC7 expression (Subramanian et al., 2020). These studies suggest that FDA-approved HDAC inhibitors can improve the development of NP-C1 to some extent in preclinical research.

In addition, other HDAC inhibitors have been reported to improve NPC1. Treatment with curcumin, a natural compound, has been shown to normalize cellular phenotypes associated with NP-C1 and prolong the survival of NP-C1 mice by regulating cytosolic calcium levels (Lloyd-Evans et al., 2008). Nicotinamide has been shown to prolong NP-C1 mouse survival and prevent oxidative stress (Marshall et al., 2017). FTY720 (fingolimod) and its active phosphorylated form (FTY720-P) act as HDAC inhibitors, increasing the expression of NPC1 and significantly reducing the accumulation of cholesterol and GSLs in human NPC1 mutant fibroblasts (Newton et al., 2017). A new synthetic HDAC inhibitor, N-hydroxy-7-(2-naphthylthio) heptanomide (HNHA), has been found to ameliorate NPC1 phenotypes such as body weight, motor function, and Purkinje cell death through autophagy induction (Jung et al., 2022). Furthermore, most HDAC inhibitors enhance the NPC1 mutant protein and promote the exit of the NPC1 protein from the ER, facilitating its delivery to late endosomes/lysosomes to stabilize cholesterol metabolism in NPC1. This process involves the refolding of the NPC1 mutant protein through changes in protein chaperones. Treatment with arimoclomol, a well-characterized heat shock protein (HSP) amplifier, or recombinant human heat shock protein 70 (rhHSP70) has been shown to reduce GSLs levels in the CNS, leading to improved cerebellar myelination and behavioral phenotypes in *Npc1* ^*−/−*^ mice (Gray et al., 2022). On the other hand, inhibiting HSP90 with several HSP90 inhibitors has been found to increase the expression of HSP70, promote the clearance of cholesterol from late endosomes/lysosomes, and reduce cholesterol storage in *NPC1* ^*I1061T*^ skin fibroblasts (Pipalia et al., 2021). These studies suggest that heat shock protein-based therapies hold promise and should be clinically evaluated for treating NP-C1.

### Other types of drugs

At present, drug development for NP-C1 predominantly centers on diminishing cholesterol buildup. Nevertheless, NP-C1 manifests several other pathological characteristics, including neuroinflammation occurs, heightened oxidative stress, disturbed ion balance, among others. Each of these pathological processes presents an opportunity for targeted drug development or combination therapy. In recent years, there has been significant advancement in the development of NPC1 drugs incorporating anti-inflammatory strategies. In a previous study, it was demonstrated that the proapoptotic tyrosine kinase c-Abl signaling is activated in *Npc1* ^*−/−*^ neurons (Contreras et al., 2016). Treatment with the c-Abl-specific inhibitor imatinib resulted in the preservation of Purkinje neurons, a reduction in general cell apoptosis in the cerebellum, improvement of neurological symptoms, and increased survival of *Npc1* ^*−/−*^ mice (Alvarez et al., 2008). Furthermore, treatment with gadolinium chloride (GdCl3) or heat-inactivated pneumococci was found to effectively reduce liver lipid accumulation and inflammation, leading to the rescue of some parameters of liver dysfunction in NP-C1 mice (Klein et al., 2018; Houben et al., 2018). In NP-C1, the necroptosis-related genes RIP3 and RIP1 are upregulated. Inhibition of necroptosis has been shown to significantly delay cerebellar Purkinje cell loss, slow the progression of neurological symptoms, and prolong survival in *Npc1* ^*−/−*^ mice (Cougnoux et al., 2016). Moreover, incorporating drugs such as ibuprofen, aspirin, metformin, and others that possess anti-inflammatory properties in combination therapy with currently known effective drugs may provide additional benefits in managing NP-C1.

Defective Ca2+ release has been associated with a number of lysosomal storage diseases (LSDs), including NP-C1. Lysosomes express big-conductance Ca2+-activated potassium (BK) channels that regulate lysosomal Ca2+ release (Cao et al., 2015). Activation of BK by NS1619 reduces lipofuscin and cholesterol accumulation in NPC1 cells in a Ca2+-dependent manner (Zhong et al., 2016). Lithium decreases STING/SREBP2 activation by reducing intracellular Ca2+ levels. Treatment with lithium has been shown to improve NP-C1 phenotypes, extend survival in *Npc1* mouse models, and enhance swallowing capacity in NP-C1 patients (Han et al., 2021; Han et al., 2023). The buildup of cholesterol in mitochondria is recognized to hinder the entry of glutathione (GSH) into mitochondria, leading to the depletion of mitochondrial GSH. However, the supplementation of GSH ethyl ester and S-Adenosyl-L-methionine has been shown to restore the mitochondrial GSH levels in the liver and brain, consequently increasing the median survival and maximum lifespan of *Npc1* ^*−/−*^ mice (Torres et al., 2017; Goicoechea et al., 2024). Furthermore, treatment with Genistein enhanced lysosomal protein expression and autophagic flux, resulting in reduced p62 levels and increased levels of LC3-II in NP-C1 patient fibroblasts (Arguello et al., 2021). These findings indicate that strategies involving anti-inflammatory actions, oxidative stress mitigation, and autophagy activation could be considered as potential treatments for NP-C1 or as complementary therapeutic approaches.

### Clinical trials

As previously noted, preclinical investigations into the efficacy of conventional small molecules for managing NP-C1 have predominantly centered on the mentioned medications. In recent years, significant strides have been made in clinical research concerning NP-C1. A review of clinical trial registrations indicates that investigations into the efficacy of HPβCD, Vorinostat, Lithium Carbonate, and other pharmaceuticals for NP-C1 are currently in progress (Table 2).

**TABLE 2:** Clinical trials for NP-C1.

ID	Drugs	Phases	Enrollment	Intervetion	Follow-up	Status	Results	Country
NCT04860960	Trappsol (R) cyclo (TM) (HPβCD)	3	94	Intravenous	5 years	Active Not recruiting	No results posted	United States
NCT03893071	Trappsol (R) cyclo (TM) (HPβCD)	1	12	Intravenous	4 years	Completed	HPβCD cleared cholesterol from the liver and improved peripheral biomarkers of cholesterol homeostasis and CNS neurodegeneration Hastings et al. (2022)	United States
NCT02939547	Trappsol (R) cyclo (TM) (HPβCD)	1	13	Intravenous	28 months	Completed	Neurologic and neurocognitive benefits were seen in most patients Hastings et al. (2022); Hastings et al. (2019)	United States
NCT02912793	Trappsol (R) cyclo (TM) (HPβCD)	1/2	12	Intravenous	4 years	Completed	Of the 9 patients who completed the study, 7 were viewed by their treating physicians as having improved to some degree at the end of the study, and 2 remained stable Hastings et al. (2019); Sharma et al. (2023)	United States
NCT04958642	Adrabetadex (HPβCD)	2/3	66	Intravenous	7 years	Terminated	Results posted in clinicaltrials.gov	United States
NCT02534844	VTS-270 (HPβCD)	2/3	56	Lumbar intrathecal	3 years	Completed	Slow disease progression; enhance neurological and neurocognitive functions; improve the physicians and caregivers Hastings et al. (2019)	United States
NCT01747135	VTS-270 (HPβCD)	1	14	Lumbar intrathecal	4 years	Completed	Slow disease progress up to 36 months post-initiation of intrathecal VTS-270 Farmer et al. (2019)	United States
NCT02124083	Vorinostat	1/2	12	Oral	32 months	Completed	Results posted in clinicaltrials.gov	United States
NCT03201627	Lithium carbonate	1	18	Oral	4 years	Completed	The mean NNSS was improved after lithium treatment. Improvement in swallowing capacity was observed in treated patients. No serious adverse events were recorded in the patients receiving lithium Han et al. (2021)	China

All information was extracted from https://www.clinicaltrials.gov/.

HPβCD has been administered to NP-C1 patients with approved Investigational New Drugs (INDs) globally since 2009. In 2015, a clinical study on the intrathecal administration of HPβCD in a 12-year-old subject with mildly symptomatic NPC demonstrated that it was generally safe and well tolerated (Maarup et al., 2015). Subsequently, open-label phase I/IIa studies of VTS-270, a formulation of HPβCD, were conducted in individuals with NP-C1 across various age groups. The research demonstrated an acceptable safety profile for VTS-270 and offered evidence of restoring neuronal cholesterol homeostasis and decelerating the progression of neurological disease (Ory et al., 2017; Farmer et al., 2019). Several case reports studying the impact of intravenous administration of HPβCD in NP-C1 children and young adults demonstrate both the safety and potential benefits of HPβCD, including an improvement in liver function and cholesterol metabolism (Hastings et al., 2022; Hastings et al., 2019; Reynolds et al., 2021). However, HPβCD is excreted rapidly from the body and has poor penetration across the human blood-brain barrier. The long-term injection of HPβCD can pose challenges due to difficulties in metabolism, potentially resulting in negative effects such as increased hearing threshold (Ward et al., 2010), inner and outer hair cells (Takahashi et al., 2016; Zhou et al., 2018; Liu et al., 2020) and significant damage to the auditory and vestibular systems (Ding et al., 2020).

Furthermore, Vorinostat and Lithium Carbonate are being utilized in clinical research for treating NP-C1. Vorinostat, as an HDACi, faces challenges in crossing the blood-brain barrier, requiring higher dosages for clinical efficacy, which may pose significant risks to patients. In comparison, Lithium Carbonate shows inferior treatment outcomes compared to HPβCD (Han et al., 2021). Therefore, to establish these drugs as effective treatments for NP-C1, further researchs are essential to overcome these obstacles and optimize combined therapeutic strategies in NP-C1 treatment.

### Combination therapy

Given the broad impact of NP-C1 on various organs and biological processes, a comprehensive treatment strategy targeting cholesterol accumulation with anti-inflammatory agents, antioxidants, and the promotion of autophagy holds great promise for addressing NP-C1. Studies have shown that non-steroidal anti-inflammatory drugs (NSAIDs) such as aspirin and ibuprofen can significantly prolong the lifespan of NPC1 mice and delay the onset of neuro-inflammation (Smith et al., 2009; Williams et al., 2014). Co-treatment with HPβCD and metformin has been found to reduce the inflammatory response in the liver, brain, and spleen of *Npc1* ^*−/−*^ mice, though it did not lead to an extension of survival time or an increase in body weight (Du et al., 2021). Furthermore, the beneficial effects of RIPK1 inhibition on *Npc1* ^*−/−*^ mice may be attributed to its role in neuroinflammation and cytokine production (Cougnoux et al., 2018b). While the combination of antioxidant drugs with HPβCD may reduce cholesterol accumulation, it does not improve NP-C1 lung pathology or alleviate cochlear damage and associated hearing loss caused by HPβCD treatment alone (Manohar et al., 2022; Erickson and Borbon, 2019). These studies show targeting inflammation in the brain represents a promising clinical intervention strategy (Table 3).

**TABLE 3:** Combination therapy in NP-C1 treatment.

Combine drugs	Models	Effects	Reference
Miglustat Aspirin Ibuprofen Vitamin C	*Npc1* ^*−/−*^ mice	Combining NSAIDs therapy prolonged the lifespan of NPC1 mice and slowed the onset of clinical signs	Smith et al. (2009)
Miglustat Curcumin Ibuprofen	*Npc1* ^*−/−*^ mice	Triple combination therapy increases the time period that maintained bodyweight and motor function and maximally delaying the onset of purkinje cell loss	Williams et al. (2014)
HPβCD metformin	*Npc1* ^*−/−*^ mice	Reduce the inflammatory response; did not extend survival time and increase the body weight	Du et al. (2021)
GSK’547 HPβCD	*Npc1* ^*−/−*^ mice NPC1 cats and patients	Slow neurological disease progression; modestly increased lifespan	Cougnoux et al. (2018b)
Probucol HPβCD	*Npc1* ^*−/−*^ mice	Does not improve lung pathology	Erickson and Borbon (2019)
Minocycline + HK-2 minocycline plus + N-acetyl cysteine HPβCD	*Npc1* ^*−/−*^ mice	Fail to attenuate the early and late phases of cyclodextrin-induced cochlear damage and hearing loss	Manohar et al. (2022)
Cyclodextrin Allopregnanolone Miglustat	*Npc1* ^*−/−*^ mice	Reveal beneficial effects on the cornea; ameliorate motor but not cognitive deficits; reduce cerebellar neurodegeneration and hepatic lipids; restore splenic cholesterol homeostasis; reduce body and brain weights	Hovakimyan et al. (2011); Hovakimyan et al. (2013); Maass et al. (2015); Ebner et al. (2018); Nesslauer et al. (2019); Holzmann et al. (2021)
Vorinostat HPβCD PEG	*Npc1* ^*−/−*^ mice *Npc1* ^*nmf164*^ mice *Npc1* ^*I1061T*^ mice	Reduce lipid storage, extend lifespan, and preserve neurological function; preserved neurites and purkinje cells, delayed symptoms of neurodegeneration, and extended mouse life span	Alam et al. (2016); Davidson et al. (2019)

Numerous studies have employed a combination therapy involving Cyclodextrin, Allopregnanolone, and Miglustat, unveiling positive effects across multiple domains. These include enhancements in corneal health, amelioration of motor deficits (although not cognitive impairments), reduction in cerebellar neurodegeneration and hepatic lipid accumulation, restoration of splenic cholesterol balance, and reductions in body and brain weights (Hovakimyan et al., 2011; Hovakimyan et al., 2013; Maass et al., 2015; Ebner et al., 2018; Nesslauer et al., 2019; Holzmann et al., 2021). Furthermore, the combination of HDAC inhibitors with HPβCD has shown promising therapeutic effects (Alam et al., 2016; Davidson et al., 2019) (Table 3).

## Gene therapy

In recent years, advancements in gene editing technology and drug delivery have propelled gene therapy for NP-C1 as a promising treatment strategy aimed at correcting the defective NPC1 gene within the patient’s body. In studies using *Npc1* ^*−/−*^ mice, researchers developed AAV9 vectors to transport the NPC1 gene under the transcriptional control of neuronal-specific (CamKII) or ubiquitous (EF1a) promoters. The results showed that treatment with AAV9-EF1a-NPC1 led to improved survival, growth, and reduced hepatic cholesterol accumulation compared to AAV9-CamKII-NPC1 (Chandler et al., 2017). This suggests that systemic AAV gene delivery may be a preferred option for NPC1 therapy.

Systemic AAV9-mediated gene therapy can significantly extend lifespan, enhance Purkinje cell survival, restore locomotor activity and coordination, prevent or alleviate neurodegeneration, reduce biochemical abnormalities, and normalize various motor function indicators through different injection approaches (Xie et al., 2017; Hughes et al., 2018; Kurokawa et al., 2021). Moreover, the meticulous selection and enhancement of AAV, as demonstrated by AAV-PHP.B shell proteins, enable the effective transfer from the periphery to the CNS. This progression results in a notably superior alleviation of disease symptoms when contrasted with a similar AAV9 vector in *Npc1* ^*m1N/m1N*^ mice (Davidson et al., 2021).

Indeed, the development of non-viral gene delivery methods is crucial in advancing gene therapy for NP-C1. A study revealed that the DNA of NPC1 is encapsulated within Trojan horse lipoproteins (THLs) that selectively target organs using monoclonal antibodies. THLs treatment reduced tissue inclusion bodies in the brain and peripheral organs but did not extend the lifespan in *Npc1* ^*−/−*^ mice (Jiang et al., 2020). Messenger RNA holds great potential as a disease-modifying treatment for NP-C1. Engineered NPC1 mRNA with optimized codons and N1-methylpseudouridine base modification has been demonstrated to correct the cholesterol transport defect in NP-C1 patient cells, confirming the promising potential of engineered mRNA in the treatment of inherited disease (Furtado et al., 2022). Compared to miglustat and CDs treatments for NP-C1, currently, there have been no reports of adverse effects resulting from the over-expression of human NPC1 through gene therapy.

## Cell-based therapy

Cell therapy is an innovative treatment approach that involves introducing healthy cells or repairing damaged cells to address a wide range of diseases, including neurodegenerative disorders. A study assessing the therapeutic impact of transplanted murine NSCs on *Npc1* ^*−/−*^ mice revealed that the implanted cells survived in the cerebellum and prolonged the lifespan of the mice. However, there was no significant improvement in body weight or ataxic symptoms, suggesting that the therapeutic effect of NSCs transplantation on NP-C1 is only partially effective (Ahmad et al., 2007). However, transplantation of human amniotic epithelial stem cells has been shown to extend the lifespan, reduce rapid weight loss, and decrease cholesterol deposition in NP-C1 mice, demonstrating promising therapeutic effects (Hong et al., 2012).

Mesenchymal stem cells (MSCs) have the ability to secrete various growth factors and contribute to tissue repair in diseases. Recent studies have shown that the transplantation of bone marrow-derived MSCs promotes the formation of neuronal networks with functional synaptic transmission, restores SphK activity, and reduces pathology in Purkinje neurons through the secretion of VEGF (Bae et al., 2005; Bae et al., 2007; Lee et al., 2014; Lee et al., 2010a). Transplantation of adipose tissue-derived MSCs has been shown to rescue Purkinje neurons, restore motor coordination, and alleviate inflammatory responses in NP-C1 mice (Bae et al., 2010). Human umbilical cord blood-derived MSCs have been demonstrated to protect against neuronal cell death and improve motor deficits by modulating neuroinflammatory conditions (Lee et al., 2010b; Seo et al., 2011). Additionally, these stem cells have the ability to suppress cholesterol synthesis and improve impaired autophagic flux in NP-C1 through the secretion of 14,15-epoxyeicosatrienoic acid (Kang et al., 2018). Recent research has suggested that conditioned medium from human menstrual blood-derived MSCs can protect against cell inflammation and apoptosis of Npc1 mutant neurons *in vitro* (Yang et al., 2023). Additionally, extracellular vesicles, which are important components of MSCs, have been demonstrated to reduce inflammation, decrease microglial and astrocyte proliferation, and modify the pathophysiological processes of NP-C1 (Van Hoecke et al., 2021). These findings offer new perspectives on the potential therapeutic use of MSCs and their extracellular vesicles in the management of NP-C1.

## Conclusion and outlook

In this review, we offer a brief overview of the current research on disease models used for NP-C1. We also conduct a comprehensive analysis of both preclinical and clinical data related to NP-C1 treatment, including traditional chemical drug therapy, gene therapy, and cell-based therapy. By systematically presenting the range of treatment options for NP-C1, this review not only consolidates current knowledge but also highlights potential directions for future research and therapeutic interventions.

NP-C1 is a rare genetic disorder primarily caused by gene mutations, characterized by the degeneration of brain nerves and damage to various organs such as the liver and spleen. Treatment challenges arise due to the necessity for therapies to effectively penetrate the blood-brain barrier to address CNS damage. Currently, the primary treatments for NP-C1 are oral miglustat and intracranial injection of HPβCD. However, both of these methods can lead to some side effects. Hence, there is an urgent requirement to develop treatment strategies utilizing innovative small molecule therapeutics. The development of gene editing technology has enabled researchers to repair mutated genes in patients within the body through precise gene delivery. This direct gene repair method holds promise as a leading approach for treating various genetic disorders. However, the heterogeneity of the *Npc1* gene among patients, coupled with the influence of diverse genetic backgrounds, leads to varying degrees of disease characteristics (Guatibonza Moreno et al., 2023). Therefore, analyzing the genetic backgrounds of individual patients is essential for the development of tailored treatments in the future (Las Heras et al., 2023). In NP-C1 patients, mutations in the *Npc1* gene often present as single nucleotide variations that result in missense mutations. Advancements in next-generation genome editors, such as base and prime editors, have demonstrated the potential to correct these mutations in NP-C1, paving the way for personalized treatment approaches. Adeno-associated viruses have shown both safety and efficacy in the body, indicating that using AAV viruses as vectors for gene therapy could be a crucial treatment option for NP-C1. Furthermore, preclinical studies on cell-based therapy have demonstrated that mesenchymal stem cell transplantation can effectively preserve Purkinje cells in NP-C1.

**FIGURE 2:**
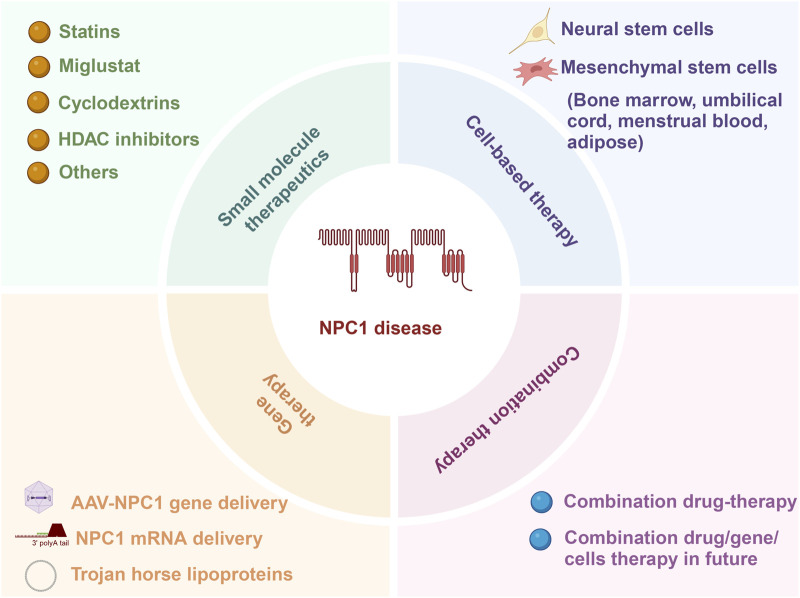
Current approaches for addressing NP-C1 encompass small molecule therapy, gene therapy, cell-based therapy, and combination therapy.

While current treatments for NP-C1 primarily focus on drug therapies and do not provide a definitive cure, the emergence of diverse treatment options offers possibilities for the comprehensive management of the condition in the future. A multifaceted approach that combines gene therapy for repairing mutated genes at the DNA level, traditional drug adjuncts to enhance cholesterol excretion and cellular phenotypes, and mesenchymal stem cell transplantation to regulate tissues and organs through vital cytokine secretion may be crucial in effectively managing this complex disorder (Figure 2). Using a combination of treatment strategies could be crucial in addressing the intricate nature of NP-C1.

## Abbreviations

NP-C1, Niemann-Pick disease type C1; LE/LY, late endosomes and lysosomes; LDL, low-density lipoprotein; CNS, central nervous system; ER, endoplasmic reticulum; iPSCs, induced pluripotent stem cells; GSLs, glycosphingolipids; CSF, cerebrospinal fluid; ROS, reactive oxygen species; NPY, Pro-neuropeptide Y; HPβCD, 2-hydroxypropyl-β-cyclodextrin; CDs, Cyclodextrins; MβCD, Methyl-β-cyclodextrin; INDs, investigational new drugs; HDACi, Histone deacetylase inhibitors; VPA, valproic acid; NSCs, neural stem cells; HSP, heat shock protein; LSDs, lysosomal storage diseases; GSH, glutathione; NSAIDs, non-steroidal anti-inflammatory drugs; MSCs, mesenchymal stem cells.
